# The Effects of Fiber Inclusion on Pet Food Sensory Characteristics and Palatability

**DOI:** 10.3390/ani5010110

**Published:** 2015-02-16

**Authors:** Kadri Koppel, Mariana Monti, Michael Gibson, Sajid Alavi, Brizio Di Donfrancesco, Aulus Cavalieri Carciofi

**Affiliations:** 1 Sensory Analysis Center, Department of Human Nutrition, Kansas State University, 1310 Research Park Drive, Manhattan, KS 66502, USA; E-Mail: briziod@ksu.edu; 2 Department of Veterinary Clinic and Surgery, College of Agricultural and Veterinarian Sciences, São Paulo State University (UNESP), Via de Acesso Prof. Paulo Donato Castellane, s/n, Jaboticabal, SP 14.884-900, Brazil; E-Mails: mariana_814@hotmail.com (M.M.); aulus.carciofi@gmail.com (A.C.); 3 Department of Grain Science and Industry, Kansas State University, Manhattan, KS 66506, USA; E-Mails: michael.gibson171@gmail.com (M.G.); salavi@ksu.edu (S.A.)

**Keywords:** dog food, extruded, fiber, palatability, sensory analysis

## Abstract

**Simple Summary:**

The results from this research indicate that fibers have an effect on extruded pet food texture and palatability. These results may help pet food companies select ingredients for successful product formulations.

**Abstract:**

The objectives of this study were to determine (a) the influence of fiber on the sensory characteristics of dry dog foods; (b) differences of coated and uncoated kibbles for aroma and flavor characteristics; (c) palatability of these dry dog foods; and (d) potential associations between palatability and sensory attributes. A total of eight fiber treatments were manufactured: a control (no fiber addition), guava fiber (3%, 6%, and 12%), sugar cane fiber (9%; large and small particle size), and wheat bran fiber (32%; large and small particle size). The results indicated significant effects of fibers on both flavor and texture properties of the samples. Bitter taste and iron and stale aftertaste were examples of flavor attributes that differed with treatment, with highest intensity observed for 12% guava fiber and small particle size sugar cane fiber treatments. Fracturability and initial crispness attributes were lowest for the sugar cane fiber treatments. Flavor of all treatments changed after coating with a palatant, increasing in toasted, brothy, and grainy attributes. The coating also had a masking effect on aroma attributes such as stale, flavor attributes such as iron and bitter taste, and appearance attributes such as porosity. Palatability testing results indicated that the control treatment was preferred over the sugar cane or the wheat bran treatment. The treatment with large sugarcane fiber particles was preferred over the treatment with small particles, while both of the wheat bran treatments were eaten at a similar level. Descriptive sensory analysis data, especially textural attributes, were useful in pinpointing the underlying characteristics and were considered to be reasons that may influence palatability of dog foods manufactured with inclusion of different fibers.

## 1. Introduction

Pet foods are manufactured with a myriad of ingredients. Mimicking human food trends, these ingredients are often of novel origin. Sensory properties of pet food products may be influenced by types of ingredients used in the formulation, added palatants, and processing factors used. Protein, grain, and fiber sources can influence the appearance, aroma, flavor and texture of extruded dog food. To understand the sensory characteristics of products manufactured with fibers of different source, eight extruded products were evaluated in this study.

Fibers are structural carbohydrates, mainly originating from plant cell walls. The energy available from fibers is limited, and because of this, fibers may be good ingredients in reduced energy diets [1]. In addition it has been found that fibers in combination with proteins help regulate satiety levels in dogs [2]. Furthermore, fibers may help regulate the digestive process and the glycemic response in dogs and cats [3,4]. Depending on the fiber type and consumed amount, different effects on nutrient digestibility and fecal formation can be promoted [4].

Previous studies have described dietary fiber effects on sensory properties of different types of foods. The different sources and amounts of dietary fibers have an effect on the texture of extruded products due to the interaction with starch [5]. The increase of insoluble fiber (mainly wheat bran) was shown to increase the hardness of extruded cereals because of reduced expansion volumes and increased density [5]. Dietary fibers enrichment in bread showed a reduction of loaf volume, with the crumbs having higher firmness and a darker appearance [6]. Other studies on bread showed that a higher content of dietary fibers resulted in smaller cells in the crumb, which in turn resulted in a more dense appearance [7]. Popov-Ralić *et al.* [8] observed that different fiber content in cookies mostly impacted the appearance of the products, in particular the color.

Most common sources of fiber in pet foods are beet pulp and cellulose. Corn fiber, fruit fibers, rice bran, and whole grains are some of the other fiber sources available for use in pet foods [3]. Full-fat rice bran was tested for palatability and digestibility in pet foods by Pacheco *et al.* [9]. These authors found that rice bran could be used in pet foods, but at no more than a 20% inclusion rate. Sa *et al.* [10] studied an enzyme treatment effect on dog foods manufactured with wheat bran. These authors found that the enzymes did not have an effect on digestibility, but wheat bran addition resulted in a larger amount of fecal matter being produced.

Understanding pet food palatability issues is not an easy task as the test animals lack the necessary linguistic capabilities. Descriptive sensory analysis by trained human panelists may provide insight into pet food palatability [11]. Descriptive sensory analysis will not tell us how the food tastes for the target species, such as dogs or cats, but will further our understanding regarding the sensory properties of the products. Sensory studies on pet foods have found that dry dog foods are generally complex products that vary in appearance, aroma, flavor, and texture [12,13]. A study that compared baked and extruded dog foods found that the pet food textures resulting from these cooking methods were significantly different [14]. However, so far no studies have been found that compare the sensory properties of pet foods to palatability or animal liking of the foods. The primary hypothesis of this study is that fiber source has an effect on sensory characteristics such as flavor and texture properties and palatability of pet foods.

The objectives of this study were to determine the following characteristics for dry dog foods formulated with a significant proportion of dietary fiber: (a) the effect of fiber on the sensory characteristics; (b) differences between coated and uncoated kibbles for aroma and flavor characteristics; (c) palatability of selected coated treatments; and (d) potential associations between palatability and sensory flavor and texture attributes.

## 2. Experimental Section

### 2.1. Diet Formulation

A basal diet containing maize and poultry by-product meal was formulated for adult dog maintenance according to the European Pet Food Industry Federation nutritional guidelines for complete and complementary pet food for cats and dogs [15]. Different types of fiber, sourced from Dilumix (Leme, Sao Paulo, Brazil), were added to this basal diet to create eight treatments as described in Table 1: control, with no fiber addition (CO); guava fruit fiber (67% insoluble dietary fiber, less than 1% soluble fiber) at the inclusion levels of 3%, 6%, and 12% (GF3, GF6, and GF12, respectively); sugarcane fiber (90% insoluble dietary fiber, less than 1% soluble fiber) with large particle size (SC1) and small particle size (SC2; Vit2be Fiber; inclusion level 9% for both treatments); and wheat bran fiber (32% insoluble dietary fiber, 1.5% soluble fiber) with large particle size (WB1) and small particle size (WB2). Ingredients were previously analyzed and the diets containing 12% guava fiber (GF12), sugarcane fiber (SC1 and SC2) and wheat bran (WB1 and WB2) were balanced to have 16% of total dietary fiber, which is a level typically used in commercial high-fiber dog diets. All fibers were added by replacing corn.

### 2.2. Grinding and Mixing

The ingredients, with the exception of fiber sources, were weighed, mixed, and ground using a hammer mill fitted with a screen size of 0.8 mm (Sistema Tigre de Mistura e Moagem, Tigre, Sao Paulo, Brazil). Fiber sources were provided already ground to desired particle sizes by the supplier (Dilumix, Leme, SP, Brazil): guava fiber—213 µm; large sugarcane fiber—395 µm; small sugar cane fiber—197 µm; large wheat bran—345 µm; small wheat bran—143 µm. These sizes were determined using laser diffraction particle size analysis [16]. The other ingredients and fiber sources were then mixed, compounding the final diet.

**Table 1:** Treatment ingredients and nutritional composition, % *.

Ingredients, %	CO	GF3	GF6	GF12	SC1	SC2	WB1	WB2
Corn grain	57.9	54.6	51.2	44.6	47.5	47.5	30.4	30.4
Chicken byproduct meal	31.3	31.6	31.8	32.3	32.6	32.6	26.1	26.1
Chicken Fat	7.0	7.0	7.3	7.4	7.2	7.2	7.6	7.6
Guava Fiber	0.0	3.0	6.0	12.0	0.0	0.0	0.0	0.0
Sugar Cane Fiber	0.0	0.0	0.0	0.0	9.0	9.0	0.0	0.0
Wheat Bran	0.0	0.0	0.0	0.0	0.0	0.0	32.0	32.0
Fish oil	0.15	0.15	0.15	0.15	0.15	0.15	0.15	0.15
Palatant	2	2	2	2	2	2	2	2
NaCl	0.5	0.5	0.5	0.5	0.5	0.5	0.65	0.65
KCl	0.5	0.5	0.5	0.5	0.5	0.5	0.5	0.5
Vitamin and Mineral mix	0.3	0.3	0.3	0.3	0.3	0.3	0.3	0.3
Choline Chloride	0.2	0.2	0.2	0.2	0.2	0.2	0.2	0.2
Mold inhibitor agent	0.1	0.1	0.1	0.1	0.1	0.1	0.1	0.1
Antioxidant agent	0.04	0.04	0.04	0.04	0.04	0.04	0.04	0.04
**Nutritional Composition in the final product (on DM-basis)**						****	****	****
Crude Protein	29.1	28.8	28.4	28.5	29.4	29.5	28.0	28.2
Crude Fat	15.3	15.9	15.2	14.6	15.0	14.7	14.5	15.5
Ash	6.4	6.0	7.0	6.1	5.8	6.2	6.5	6.4
Crude Fiber	1.9	3.0	3.9	4.7	4.6	4.2	3.1	3.7
Dietary fiber	8.0	9.9	11.9	15.7	16,1	16.3	16.9	16.7
Starch	40.2	38.7	35.7	34.6	35.6	34.8	32.4	32.4
Moisture	5.9	6.8	6.0	7.3	5.4	5.3	5.6	5.5

* CO-Control, GF3—3% guava fiber, GF6—6% guava fiber, GF12—12% guava fiber, SC1—sugar cane fiber large grind, SC2—sugar cane fiber small grind, WB1—wheat bran fiber, large grind, WB2—wheat bran fiber, small grind.

### 2.3. Extrusion

The diets were extruded in a single screw extruder (MEX 250, Mazoni, Campinas, Brazil), with a processing capacity of 250 kg/h. Each food was processed separately on two different days for replicates. The processing conditions were not changed for any treatment in order to isolate the influence of fiber. Four samples were collected per diet each day and pooled. Pooled samples from both days were combined into one batch per diet for the sensory and palatability studies. For each diet, suitable amounts of samples were selected for coating for palatability and sensory tests from the same consolidated batch as the uncoated samples. A pre-conditioner was used to treat the diets with steam and water prior to extrusion. The pre-conditioner residence time was approximately 3.5 min, and downspout temperature was 82.2–85.7 °C. The extruder screw speed was 465 rpm, and the die open area was 15.9 mm^2^/ton/h. Extruder die temperature ranged between 118.7–130.3 °C and die pressure between 52.45–70.6 bar. After extrusion, the kibbles were dried in a forced air dryer at 105 °C for 30 minutes. The dried kibbles were coated in a tumble system, receiving first the poultry fat and then the commercial palatant D’TECH 6L (SPF, Descalvado, São Paulo, Brazil) at 2% of the ingredients. The fish oil in the formulation was mixed with dry ingredients before extrusion.

### 2.4. Descriptive Sensory Analysis

#### 2.4.1. Panelists

Five highly trained panelists from the Sensory Analysis Center, Kansas State University (Manhattan, KS, USA) participated in this study. All of the panelists had completed 120 h of general descriptive analysis training with a variety of food products. The training included techniques and practice in attribute identification, terminology development, and intensity scoring. Each of the panelists had more than 1000 h of testing experience with a variety of food products. For this project, the panelists received further orientation on dried dog food using samples that may or may not be included in the study. Panels of similar size and training have been reported in other recent research [8,9,10].

#### 2.4.2. Sample Presentation and Evaluation

The panelists evaluated both uncoated (no poultry fat and no palatant added) and coated (poultry fat and palatant added) kibbles to determine the effect of coating and palatant on kibble flavor.

Evaluation of uncoated kibbles (*n* = 8: CO, SC1, SC2, WB1, WB2, GF3, GF6, and GF12) was conducted during seven 1.5 h sessions. Evaluation of coated kibbles (*n* = 5: CO, SC1, SC2, WB1, and WB2) was conducted during five 1.5 h sessions.

Each sample was served in a ~100 mL plastic cup for appearance, texture, and flavor evaluation, and in a medium snifter covered with a watch glass for the evaluation of aroma. The amount of product in the snifter was 3 g. Samples were prepared 30 min prior to the testing and were coded with three-digit random numbers.

All of the uncoated samples were evaluated in a randomized order in duplicate for appearance, texture, flavor, and aroma using attributes selected from a lexicon developed for this product category by Di Donfrancesco *et al.* [12] and used by Koppel *et al.* [13]. Barnyard, brothy, toasted, grain, vitamin, stale, egg, oxidized oil, cardboard, liver, fish, metallic, and dusty/earthy aroma and flavor were evaluated in all samples. In addition sour, salty, sweet, and bitter taste and aftertaste and barnyard, vitamin, stale, oxidized oil, cardboard, liver, fish, and metallic aftertaste attributes were evaluated. Brown color intensity, porous, grainy, flecks (yes/no), and fibrous appearance characteristics and cohesiveness of mass, fracturability, hardness, initial crispness, fibrous and gritty texture attributes were evaluated. A total of two 1.5 h sessions were held for orientation purposes and five 1.5 h sessions for the evaluation phase of the samples.

The coated samples (*n* = 5) were evaluated in a randomized order in duplicate for aroma, flavor, and appearance attributes only. The appearance attributes evaluated were: brown, porous, grainy, fibrous, and flecks. The aroma attributes were: barnyard, brothy, toasted, grain, vitamin, egg, sour aromatics, oxidized oil, cardboard, dusty/earthy, iron, and hay-like. The flavor attributes were: barnyard, broth, toasted, grain, vitamin, egg, sour, salt, bitter, sweet, oxidized oil, cardboard, liver, fish, dusty/earthy, metallic, iron, and hay-like. A total of two 1.5 h sessions were held for orientation purposes and three 1.5 h sessions for the evaluation phase of the coated samples.

For the evaluation, a numeric scale of 0–15 with 0.5 increments where 0 represented none and 15 extremely high was applied to each attribute to provide a measure of intensity. Each panelist individually assigned intensities to the attributes present in the sample according to their perception of the appearance, aroma, flavor, and texture references included in the lexicon. The panelists were provided with a definition sheet with the list of attributes and their definitions as well as reference materials for each attribute according to Di Donfrancesco *et al.* [12].

The panelists were asked to chew one kibble for flavor and texture evaluation. The panelists were instructed to expectorate samples after evaluation. Panelists were provided with apple slices, unsalted crackers, purified water, and toothbrushes for palate cleansing in between the evaluations. The testing room was at 21 ± 1 °C and 55% ± 5% relative humidity.

### 2.5. Palatability Testing

#### Palatability Testing Procedure

The tests were performed in Panelis, Diana Group (Descalvado, São Paulo, Brazil). Palatability was measured for the five fiber treatments (CO, SC1, SC2, WB1, and WB2) and with the coated kibbles only, using the two-pan method on two meals in one day, each test with 38 dogs in individual kennels [17]. The combinations of the samples were CO × SC1, CO × WB1, SC1 × SC2, and WB1 × WB2. In the morning after a 12 h fast the dogs received two pans, each containing one of the experimental foods, and were allowed to eat for 30 min. The position of the food pans was alternated at the evening meal. The amount of food offered in each pan surpassed the consumption capacity of the animal to ensure there would be leftovers to measure. After 30 min the pans were removed, the remains weighed and consumption rate was calculated (Equation (1)). Due to the large differences in body weights the results were calculated as relative consumption of each diet, and the mean intake of the two meals for each dog was compared.

(1) $$\text{Relative}\;\text{consumption}\;(\%)=\frac{\text{Food}\;\text{A}\;\text{consumption}}{\text{Food}\;\text{A}\;\text{consumption}+\text{Food}\;\text{A}\;\text{consumption}}\times 100$$

### 2.6. Data Analysis

Significant differences (*p* < 0.05) among the uncoated kibble treatments and coated kibble treatments sensory properties were determined using SAS Glimmix procedure and Fisher’s protected Least Significant Difference (LSD) (Version 9.3, SAS Institute Inc., Cary, NC, USA). Principal Components Analysis was conducted for the significantly different appearance, texture, aroma, and flavor attributes of Control, WB1, WB2, SC1, and SC2 using XLStat version 2014.1.08 (Addinsoft, New York, NY, USA). Dog palatability data were statistically evaluated using Analysis of Variance (ANOVA), using the SAS software (Version 9.3, SAS Institute Inc.).

## 3. Results and Discussion

### 3.1. Descriptive Sensory Analysis

#### 3.1.1 Uncoated Treatments

The main contributing attributes to aroma for the uncoated treatments were barnyard, toasted, grainy, stale, and cardboard [18]. These attributes were higher in intensity than other attributes, but were not necessarily different among treatments. The main contributing attributes to flavor and taste for these dog foods were barnyard, toasted, grainy, vitamin, stale, sour, salty, bitter, dusty/earthy, oxidized oil, cardboard, liver, and metallic. The highest intensity was noted for bitter taste and aftertaste (averages 6.70–8.90).

The uncoated treatments were not significantly different (*p* > 0.05) in brown color, cohesiveness of mass, hardness, vitamin aftertaste, sour aftertaste, salty aftertaste, cardboard aftertaste, liver aftertaste, metallic aftertaste, barnyard flavor, broth flavor, grain flavor, vitamin flavor, sour taste, salty taste, sweet taste, cardboard flavor, liver flavor, fish flavor, metallic flavor, iron flavor, barnyard aroma, broth aroma, toasted aroma, grainy aroma, vitamin aroma, stale aroma, eggy aroma, cardboard aroma, liver aroma, fishy aroma, or iron aroma.

**Table 2:** Average texture, appearance, aroma, flavor, and aftertaste attributes intensity scores for uncoated treatments. Significantly different attributes showed only (*p* < 0.05).

Attribute	Treatment							
	CO	GF3	GF6	GF12	SC1	SC2	WB1	WB2
Fracturability	7.03 ^a^	6.60 ^ab^	6.73 ^ab^	6.37 ^bc^	5.67 ^d^	5.63 ^d^	7.17 ^a^	5.90 ^cd^
Initial Crispness	10.47 ^a^	10.47 ^a^	10.43 ^a^	9.33 ^b^	8.87 ^bc^	8.73 ^c^	10.33 ^a^	9.17 ^bc^
Fibrous	1.63 ^de^	3.03 ^bc^	0.70 ^e^	2.07 ^cd^	9.20 ^a^	4.07 ^b^	1.93 ^cde^	3.10 ^bc^
Gritty	4.23 ^ab^	4.23 ^ab^	4.87 ^a^	4.03 ^b^	3.37 ^c^	4.13 ^b^	3.97 ^bc^	3.87 ^bc^
Porous Appearance	5.00 ^bc^	5.27 ^abc^	6.13 ^a^	5.80 ^ab^	4.60 ^c^	2.93 ^d^	6.17 ^a^	6.23 ^a^
Grainy Appearance	2.00 ^bcd^	2.10 ^bc^	2.60 ^ab^	1.93 ^cd^	2.90 ^a^	1.47 ^d^	2.13 ^bc^	1.90 ^cd^
Fibrous Appearance	1.10 ^c^	1.00 ^c^	0.60 ^c^	1.07 ^c^	6.17 ^a^	2.40 ^b^	0.80 ^c^	1.30 ^c^
Oxidized Oil Aroma	2.13 ^ab^	1.87 ^b^	2.37 ^a^	2.37 ^a^	1.77 ^b^	2.00 ^ab^	1.83 ^b^	1.73 ^b^
Dusty/Earthy Aroma	2.30 ^bc^	2.23 ^bc^	2.50 ^ab^	2.60 ^ab^	2.77 ^a^	2.50 ^ab^	2.07 ^c^	2.43 ^abc^
Stale Flavor	2.77 ^b^	2.73 ^b^	3.03 ^ab^	3.07 ^ab^	3.00 ^ab^	3.23 ^a^	2.83 ^b^	2.80 ^b^
Eggy Flavor	1.10 ^a^	1.33 ^a^	1.23 ^a^	1.17 ^a^	0.40 ^b^	0.93 ^a^	1.40 ^a^	1.00 ^a^
Bitter Taste	7.57 ^b^	6.70 ^c^	8.07 ^ab^	8.33 ^a^	7.93 ^ab^	8.30 ^a^	7.37 ^bc^	7.93 ^ab^
Dusty/Earthy Flavor	2.13 ^c^	2.23 ^c^	2.53 ^abc^	2.73 ^ab^	2.77 ^a^	2.27 ^c^	2.33 ^bc^	2.43 ^abc^
Oxidized Oil Flavor	2.24 ^b^	2.23 ^b^	2.80 ^a^	2.80 ^a^	2.20 ^b^	2.20 ^b^	2.20 ^b^	2.23 ^b^
Barnyard aftertaste	2.87 ^d^	3.17 ^bcd^	3.47 ^ab^	3.67 ^a^	3.43 ^ab^	3.00 ^cd^	3.20 ^bcd^	3.27 ^bc^
Stale aftertaste	2.67 ^c^	3.00 ^ab^	3.10 ^ab^	3.17 ^a^	2.83 ^bc^	3.00 ^ab^	2.67 ^c^	2.90 ^abc^
Bitter aftertaste	8.07 ^bcd^	7.93 ^cd^	8.70 ^ab^	8.90 ^a^	8.47 ^abcd^	8.63 ^ab^	7.87 ^d^	8.53 ^abc^
Sweet aftertaste	0.20 ^ab^	0.03 ^bc^	0.00 ^c^	0.27 ^a^	0.03 ^bc^	0.00 ^c^	0.13 ^abc^	0.00 ^c^
Oxidized oil aftertaste	2.20 ^b^	2.33 ^b^	2.57 ^ab^	2.90 ^a^	2.33 ^b^	2.30 ^b^	2.23 ^b^	2.27 ^b^
Fish aftertaste	1.27 ^ab^	1.03 ^b^	1.80 ^a^	1.80 ^a^	1.40 ^ab^	1.10 ^b^	1.77 ^a^	1.27 ^ab^
Iron aftertaste	1.77 ^c^	2.33 ^abc^	2.37 ^abc^	2.87 ^a^	2.30 ^abc^	2.43 ^ab^	2.17 ^bc^	1.93 ^bc^

Appearance attributes porous, grainy, and fibrous were significantly different (*p* < 0.05) among the uncoated treatments (Table 2). The two wheat bran fiber diets, WB1 and WB2, had the highest scores for porous attribute, while the small particle size sugar cane (SC2) has the lowest. Sugar cane large particle size (SC1) had the highest fibrous (more than three point-scales from the second highest score) and grainy appearance level while sugar cane small particle size (SC2) had the lowest level for grainy attribute.

Off-notes oxidized oil and dusty/earthy were significantly different for aroma and flavor among the uncoated treatments. Samples GF6 and GF12 showed the highest oxidized oil aroma and flavor level. Stale, egg, and bitter were significantly different in flavor. Samples GF12 and SC2 were the most bitter while GF3 was the least bitter sample. The samples varied in barnyard, stale, bitter, sweet, oxidized oil, fish, and iron aftertaste. Sample GF12 had the highest bitter aftertaste and was the highest in stale, barnyard, sweet, oxidized oil, and fish aftertaste.

Fracturability, initial crispness, fibrous, and gritty attributes differentiated the uncoated treatments in texture. The largest difference in texture was found in the fibrous attribute. Sample SC1 was more than five points higher than the second highest score among the treatments. For other texture attributes the treatments showed smaller differences.

Least significant differences are shown with superscript letters following the average intensity scores. Letters that are the same for a treatment attribute in a row are not significantly different (*p* > 0.05).

The results indicate that fiber source as well as fiber amount and particle size have an influence on the sensory properties of extruded dog food. For aroma and flavor, the overall differences were not too large across the treatments. Bitter taste and stale and iron aftertaste were examples of flavor attributes that differed among treatments. Sample GF12 (together with sample SC1) showed the highest scores for most of the aftertastes (including off-notes) and the highest bitterness. GF12 had the least particle size and also one of highest levels of total dietary fiber among all treatments, which might be factors contributing to highest intensity scores for various attributes described above. On the other hand, GF3 had the least dietary fiber level, which was probably the reason for its lowest bitter score. Martin *et al.* found that dietary fibers enriched breads were characterized by a higher bitterness together with higher cereal and toasted notes [6]. Sugar cane fibers with large particle size (sample SC1) seemed to influence fibrous texture more than any other type of fibers. Sugar cane samples also showed the lowest fracturability and initial crispness (texture) levels, possibly because the length/size of sugar cane fibers was higher than other fibers, leading to a strengthening effect. This was probably also the reason for the above observation related to fibrous texture and was reflected in appearance as well. SC1 showed the highest scores across the sample set for fibrous and grainy appearance attributes. Treatments with sugar cane fiber (SC1 and SC2) also had the least porous appearance. Interestingly, among all fiber sources, wheat bran appeared to have the least impact on most sensory attributes. WB2 showed lower fracturability and initial crispness than WB1. Smaller particle size has been reported previously to result in lower levels of hardness in the case of extruded cereals [5].

#### 3.1.2. Coated Treatments

The main contributing attributes to aroma of the coated treatments were barnyard, brothy, toasted, grainy, dusty/earthy, cardboard, and hay-like [18]. The main contributing attributes to flavor and taste of these dog foods were barnyard, brothy, toasted, grainy, vitamin, sour, salty, bitter, dusty/earthy, cardboard, and hay-like. The highest intensity was noted for bitter taste (averages 5.94–6.56). Commercial pet foods are typically coated with a palatant; in general, commercial dry dog foods exhibit sensory characteristics such as barnyard, brothy, brown, grain, soy, vitamin, oxidized oil, cardboard, and stale [12]. These attributes were similar to the main flavor and taste attributes detected in experimental dry dog foods in this study.

After coating with poultry fat and a palatant there were few significant differences (*p* < 0.05) found among the treatments (Table 3). Brown and porous appearances were the attributes that varied most among the treatments. Wheat bran treatments WB1 and WB2 were significantly darker brown than sugar cane treatments. The control sample was the lightest brown in appearance. Porous appearance was more apparent for the control and WB2 samples. Toasted aroma was found to be less intense in sample SC2, while dusty/earthy aroma was less intense in sample WB1. Sample WB1 was also less intense in cardboard flavor.

**Table 3:** Average appearance, aroma, and flavor attribute intensity scores for coated treatments. Significantly different attributes showed only (*p* < 0.05).

Attribute	Treatment				
	CO	SC1	SC2	WB1	WB2
Brown Appearance	7.19 ^c^	7.69 ^ab^	7.50 ^bc^	7.94 ^a^	7.94 ^a^
Porous Appearance	2.44 ^a^	1.88 ^b^	1.81 ^b^	1.25 ^c^	2.38 ^a^
Toasted Aroma	3.38 ^a^	3.00 ^a^	2.56 ^b^	3.13 ^a^	3.19 ^a^
Dusty/earthy Aroma	2.94 ^a^	2.94 ^a^	2.88 ^a^	2.44 ^b^	3.06 ^a^
Cardboard Flavor	3.19 ^a^	3.13 ^a^	2.81 ^a^	2.31 ^b^	2.81 ^a^

Least significant differences are shown with superscript letters following the average intensity scores. Letters that are the same for a treatment attribute in a row are not significantly different (*p* > 0.05).

#### 3.1.3. Coating Effect on Sensory Properties

**Figure 1:**
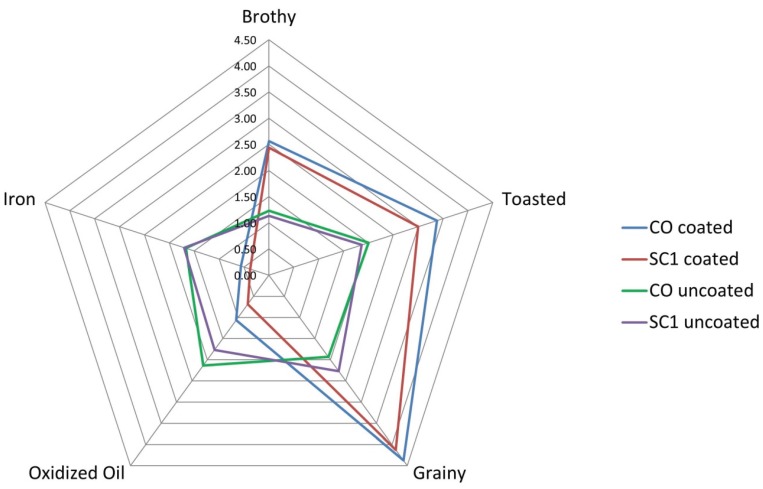
Poultry fat and palatant effect on kibble aromatics. Only the Control (CO) sample and Sugar Cane large particles (SC1) samples are shown.

**Figure 2:**
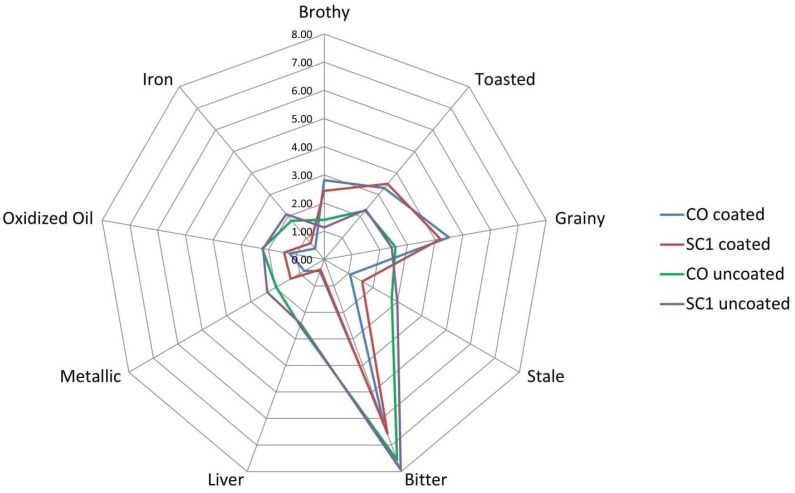
Poultry fat and palatant effect on kibble flavor. Only the Control (CO) sample and Sugar Cane large particles (SC1) samples are shown.

Poultry fat and palatant effects on appearance, aroma, and flavor attributes among the uncoated and coated treatments were noticed. The coated treatments were less porous in appearance, albeit more intense in brown color. The aromatics of the treatments changed after coating with the poultry fat and palatant (Figure 1). Specifically, stale, liver, and fish aromatics were present in the uncoated kibbles, but were not detected in the coated kibbles. Similarly, sour aromatics and hay-like aroma was detected only in the coated kibbles. Brothy, grainy, and toasted attributes increased in intensity and oxidized oil and iron aromatics reduced in intensity after coating with poultry fat and palatant. The flavor of the treatments changed after coating with the poultry fat and palatant (Figure 2). Fish flavor was not detected in the coated kibbles, while hay-like flavor was detected in the coated kibbles. Brothy, toasted, and grainy flavor increased after coating the kibbles. Stale, liver, oxidized oil, metallic, and iron flavor and bitter taste decreased after coating the kibbles. It was apparent that coating had a masking effect on appearance attributes such as porosity, aroma attributes such as stale, and flavor attributes such as iron and bitter taste.

### 3.2. Palatability

According to the palatability testing results some of the treatments were more palatable than others (Table 4). For example the CO treatment was eaten more than SC2 or WB2, thus implying that fiber addition decreased the palatability. This is not a surprising result, given the negative attributes associated with fiber in general, including hardness and bitterness. A study conducted by Sa *et al.* [10] looked at using wheat bran in dog foods. These authors found that the dogs in the experiment actually consumed the negative control sample less than the wheat bran treatments, however, this was not a preference test. Sample WB1 was eaten more than WB2, and SC1 was eaten more than SC2. These results indicated that both fiber addition and the size of the fiber particles might have an influence when determining whether the diet is palatable or not. A full set of comparisons among the treatments would be needed in order to determine an order of preference.

**Table 4:** Palatability testing results.

Comparison	Treatment	First Choice (%)	Food intake (%)
CONTROL *versus* SC2	Control	70 **	80 ***
	SC2	30	20
CONTROL *versus* WB2 *	Control	75 **	88 ***
	WB2	25	12
SC1 *versus* SC2	SC1	87 ***	79 ***
	SC2	13	21
WB1 *versus* WB2	WB1	24	55
	WB2	76 **	45

* This test was not validated due to underconsumption of the treatments by the dogs. ** difference between groups (*p* < 0.05). *** difference between groups (*p* < 0.01).

### 3.3. Potential Effect of Sensory Properties on Palatability

Further inspection of the treatments sensory properties and palatability testing results indicated that the texture properties of CO and WB1 were similar in fracturability, initial crispness, and fibrous attributes. The CO sample was preferred over both SC2 and WB2. The WB1 sample was only compared to WB2 sample, and the results showed a somewhat higher, but not significantly higher intake for WB1 sample (Table 4). These results indicate that texture properties may have an influence on dry pet food palatability, and higher intensity of attributes such as fracturability and initial crispness might lead to greater palatability.

Most variability was detected in the fibrous texture attribute. This was probably caused by the different fiber sources used in the formulation. The CO and WB1 samples were similarly low in this attribute, while WB2 and SC2 were higher, and SC1 the highest (9.20, Table 2). The fibrous attribute was defined as “the perception of fibers and filaments in the product after three to five chews”. Most dogs do not chew the food too extensively, but the fibrous texture is likely to have an impact on the mouthfeel of the food. Based on the results from palatability testing, it seems that fibrous mouthfeel may have an effect on pet food preference. When considering that SC1 was preferred over SC2, however, and that SC1 was highest in fibrous characteristics, the role of this attribute in determining palatability should be studied further.

There were only three aroma and flavor attributes that were significantly different among the coated treatments (toasted and dusty/earthy aroma and cardboard flavor). Toasted aromatics were the highest in the CO sample, and lowest in SC2 sample. As the CO was eaten more in the comparisons, and SC2 was eaten less in the comparisons, this attribute may be important in determining palatability of dry pet food. The texture effect was expected to be more prominent in these treatments, as the palatant amount and formulation was kept constant for each of the treatments. Nevertheless these differences in aroma and flavor were likely to be the result of the ingredient differences and may have influenced palatability as well.

In a comparison between WB1 and WB2, WB2 was most often the first choice for dogs. This did not result in this sample being the most eaten, though. Treatment WB2 showed a higher dusty/earthy aromatics level, and this could be a factor that attracted the dogs before they actually started eating. A flavor note that was different between WB1 and WB2 was cardboard, and WB2 had a higher intensity of this flavor. This may be one of the reasons for preference of WB1 once dogs started eating the treatment.

**Figure 3:**
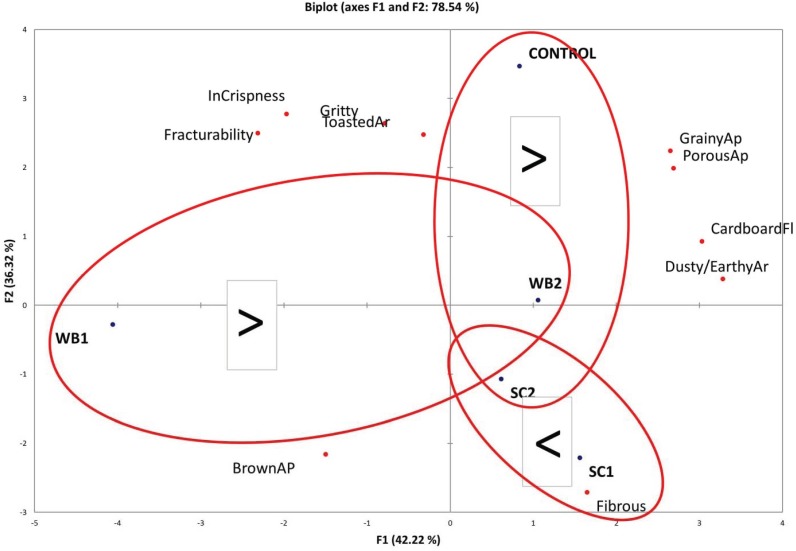
Principal Components Analysis of treatments for the significantly different appearance (Ap) texture, aroma (Ar), and flavor (Fl) attributes. Texture attributes were included based on the uncoated treatment descriptive sensory analysis results. In Crispness—Initial crispness. The < and > symbols indicate the direction of the palatability test for the comparisons of samples within the ovals.

A Principal Components Analysis (PCA) was conducted in order to visualize the main differences between the treatments and to relate these to dog preference testing results. A total of 78.54% of variability among the treatments was explained by the two first Principal Components (Figure 3). According to the PCA analysis in the comparison of treatments WB1 and WB2, porous appearance and cardboard flavor and dusty/earthy aromatics may have influenced palatability. The comparison between CO and WB2 or SC2 resulted in the CO treatment being more palatable. This may have been caused by higher toasted aromatics, grittiness, initial crispness, or fracturability. The comparison between SC1 and SC2 resulted in the SC1 treatment being more palatable. This may have been caused by the more fibrous texture of treatment SC1.

There are some limitations to this research. The first and most obvious is the fact that human senses are different from a dog’s senses. This study was not trying to claim that the senses of a human and those of a dog are similar. Rather the study was trying to understand if there were any sensory attributes that the human panelists could distinguish in dog foods that may help indicate direction of preference in a palatability test. While the results from this study may not necessarily fully answer that question, at least as far as fiber sources in pet foods are concerned, some indication of the reasons behind higher liking were detected.

The second limitation lies in the palatability test and the amount of information collected with this test. Typically, characteristics like first choice and food intake are collected. During an eating situation, dogs, just like humans, are likely to indicate their liking with other bodily cues, such as wagging the tail, increased heart rate or respiration rate, pupil dilation, licking of the mouth, or other indications. A study conducted by van den Bos *et al.* [19] concluded that cats might indicate their liking or disliking of foods with some behavioral, facial or tongue movements. Further research is needed to understand what cues may indicate liking in dogs. In addition, the two-bowl testing situations are somewhat forced in their nature. The dogs have two choices of food to choose from, and there is no guarantee that either of the foods is more palatable. Since the dogs are likely to be hungry, they will probably eat either or both of the foods, but this may not necessarily show that either of the foods is liked more.

Furthermore, the use of kennel dogs may provide different results than the use of actual pets that live with their owners. This has been shown by Smith *et al.* [20] in a comparison of laboratory and pet dogs in their preferences for dog foods that varied in flavor. In the current study, the focus was on texture attributes, as the flavor variation was kept at a minimum by using the same palatant on all treatments. A comparison of different textures of dog foods has been conducted by Kitchell *et al.* [21], however this comparison was among moist, canned, and dry foods. A preference of canned and moist foods over dry foods was found in that study. No studies were found that compared dog preference for different dry food textures.

## 4. Conclusions

The results of this study indicated that fibers (type or quality, particle size, and amount) have an influence on sensory characteristics of extruded dog food. The texture of treatments was most influenced by the different fibers, and sugar cane fibers with large particle size influenced the fibrousness of treatments more than other fiber sources. Moreover, the higher length of sugar cane fibers, causing a strengthening effect, could have played a role in the lowest score in fracturability and initial crispness. Fibers seemed to influence aroma and flavor properties; the treatment with the highest amount of guava fibers was the most bitter and had most aftertastes, including off-notes such as stale and oxidized oil. The coating process showed to be fundamental in reducing or eliminating off-notes both in flavor and aroma, and also altering the appearance. Moreover it increased notes such as brothy, grainy, and toasted in the samples and even added new characteristics not present in the uncoated samples such as sour taste and hay-like aroma and flavor.

Palatability testing results indicated that the control treatment was preferred over treatments containing small particle size of both sugar cane and wheat bran fibers. Product hardness, bitter taste and other off-notes associated with fibers might have been contributing factors. When the same fiber sources where compared, a preference for large particle size was shown for the food intake. Wheat bran fibers with small particle size showed to be preferred as first choice but not in regard to food intake.

Results showed that texture characteristics such as fracturability, initial crispness, and grittiness, together with aromas such as toasted may play a main role in increasing the palatability. Moreover, different results among first choice and food intake for a particular source of fiber may be helpful to indicate what kind of aromas may be more attractive for dogs, and what kind of flavors may cause them prefer a treatment that was perhaps not chosen in the first moment.

Further future studies on the subject may clarify the influence of different fibers on palatability further and give a better understanding of the relationship between sensory properties of extruded dog food and acceptance by dogs.
